# Effects of Methionine Supplementation in Low-Protein Diets on Growth Performance, Fur Quality, Blood Indices, and Intestinal Microbiota of Blue Foxes (*Vulpes lagopus*) During the Fur-Growing Period

**DOI:** 10.3390/ani16040573

**Published:** 2026-02-12

**Authors:** Huali Shi, Sibo Cheng, Zhongbo Sun, Chengkai Yang, Xinyan Cao, Chongshan Yuan, Aiwu Zhang

**Affiliations:** College of Animal Science and Technology, Jilin Agricultural University, Changchun 130118, China; 15584982356@163.com (H.S.); 15754473057@163.com (S.C.); 18143065538@163.com (Z.S.); 13504757497@163.com (C.Y.); xinyan_99@163.com (X.C.)

**Keywords:** blue foxes, fur-growing phase, low-protein diets, methionine, intestinal microbiota

## Abstract

This study evaluated the effects of supplementing methionine to a low-protein diet on nutrient digestibility, nitrogen (N) metabolism, growth performance, serum biochemical parameters, fur quality, and intestinal microbiota composition in blue foxes (*Vulpes lagopus*) during the fur-growing period. Fifty 17-week-old blue foxes were randomly allocated to five experimental groups (*n* = 10 per group). The control group received a diet containing 28% crude protein (CP), while the experimental groups were fed a 22% CP diet supplemented with 0%, 0.35%, 0.55%, or 0.75% methionine on a dry matter (DM) basis, designated as M0, M1, M2, and M3, respectively. Results demonstrated that the final body weight (FW), total weight gain (TW), and average daily gain (ADG) of the M3 group were comparable to the control group (*p* > 0.05). Methionine supplementation significantly enhanced fur quality and stimulated hair follicle development (*p* < 0.05). Although the reduction in dietary protein level led to decreased N intake and fecal N excretion, the M2 and M3 groups exhibited significantly higher N retention compared to the control, M0, and M1 groups (*p* < 0.05). Regarding nutrient digestibility, the M2 and M3 groups showed higher DM digestibility (*p* < 0.05), while the M3 group maintained organic matter (OM) digestibility comparable to the control group (*p* > 0.05). The highest CP digestibility was observed in the M3 group (*p* < 0.05). Additionally, ether extract (EE) digestibility was significantly improved in the methionine-supplemented groups (M1–M3) relative to the control (*p* < 0.05). Serum analysis revealed dose-dependent increases in total protein (TP), albumin (ALB), and high-density lipoprotein (HDL) concentrations in the M2 and M3 groups. Conversely, low-density lipoprotein (LDL) levels were elevated in these groups compared to the control and M0 groups (*p* < 0.05). Liver function parameters were also significantly improved in the M2 and M3 groups (*p* < 0.05). Furthermore, methionine supplementation enhanced the diversity and richness of the intestinal microbiota and altered its composition at the phylum and genus levels. In conclusion, supplementing low-protein diets with methionine can maintain growth performance, improve fur quality, enhance nutrient utilization efficiency, and support intestinal microbiota homeostasis in blue foxes. The optimal supplementation level is 0.75%, resulting in a total dietary methionine concentration of 1.1% on a DM basis.

## Simple Summary

Reducing the crude protein (CP) level in animal feed can lower feed costs and mitigate nitrogen (N) emissions. However, this reduction concurrently decreases the supply of essential amino acids, which can compromise animal growth, development, and production performance. Methionine, as the first limiting amino acid for fur-bearing animals, plays a pivotal role in the hair follicle development and fur quality of blue foxes (*Vulpes lagopus*) during the winter fur-growth period. A deficiency in methionine directly impairs fur quality. Therefore, this study aimed to investigate the effects of reducing dietary protein content while supplementing with different levels of methionine on the growth performance, health status, and fur quality of blue foxes during this critical phase. The results demonstrated that methionine supplementation not only maintained normal growth and fur quality but also enhanced nutrient digestibility, N utilization efficiency, and serum biochemical parameters. Furthermore, methionine supplementation increased both the richness and diversity of the intestinal microbiota. These findings indicate that a moderate reduction in dietary protein, when combined with methionine supplementation, can sustain animal production performance and health, improve feed efficiency, reduce N emissions, and promote sustainable breeding practices.

## 1. Introduction

The blue fox (*Vulpes lagopus*) is a natural color morph of the Arctic fox. As a domesticated carnivorous species, it undergoes seasonal molting commencing in late September, followed by a phase of rapid pelage growth. During this critical physiological stage, the ingestion of high-quality protein is indispensable for sustaining optimal growth performance, pelage quality, and overall health status [1,2]. Traditional blue fox diets primarily consist of animal-derived or conventional plant-based protein sources, such as fish meal, soybean meal, and fresh fish. This reliance on traditional protein sources often leads to substantial feeding costs. To reduce these expenses, the inclusion of plant proteins in diets has been gradually increased. Concurrently, advances in long-term genetic selection and marker-assisted breeding have significantly enhanced the physiological adaptability of blue foxes to plant-based ingredients. However, current evidence indicates that the overall digestibility of plant proteins in fur-bearing animals remains markedly lower than that of animal-derived proteins [3,4]. The lower digestibility often results in imbalances in essential amino acid profiles. Methionine has been identified as the primary limiting amino acid and is frequently deficient in these diets [5]. In commercial farming practices, producers often increase the inclusion of plant proteins to elevate CP levels in animal diets, aiming to compensate for amino acid deficiencies. However, this strategy not only intensifies the hepatic metabolic burden associated with degrading excess amino acids but also markedly increases N excretion [6,7], thereby elevating environmental nitrogen pollution risks. In modern animal husbandry, there is a demand for reducing dietary protein levels while providing animals with precise amino acid nutrition. This approach advocates targeted supplementation of limiting amino acids in a dose-controlled manner based on the metabolic requirements specific to different physiological stages, with the goal of balancing production performance, animal health, and environmental sustainability [8,9]. Therefore, for blue foxes during the fur development phase, scientifically integrating plant protein sources while optimizing their limiting amino acid profiles represents a more effective strategy than simply increasing the CP content of plant-based feeds. This integrated approach better supports the diverse nutritional needs associated with growth, tissue metabolism, and hair follicle development.

During the critical fur growth stage, blue foxes require extensive synthesis of structural hair proteins, primarily composed of keratin. As the sole sulfur-containing essential amino acid in animals, methionine plays a key regulatory role in protein synthesis and the production of various bioactive compounds, and it serves as the primary limiting amino acid for growth and fur development in fur-bearing animals [5]. Existing research indicates that methionine significantly regulates hair follicle morphogenesis and enhances hair follicle density [10,11]. Furthermore, minks fed a low-protein diet supplemented with 0.6% Met exhibited superior ADG and feed-to-gain ratio (F/G) compared to those in the high-protein group, with the highest daily N retention [12]. Concurrently, studies on blue foxes have also demonstrated that reducing the dietary CP level can improve N utilization efficiency [13]. However, systematic research on the critical fur growth period remains limited, and the optimal methionine supplementation level in low-protein diets has not yet been established. Therefore, this study aims to systematically evaluate the effects of graded methionine supplementation on growth performance, fur quality, nutrient digestibility, serum biochemical parameters, and intestinal microbiota of blue foxes during the fur growth period. The objective is to determine the optimal methionine supplementation level in low-protein diets and to provide scientific evidence for optimizing low-protein feeding strategies in fur-bearing animal production.

## 2. Materials and Methods

### 2.1. Animals, Diets, and Management

A total of 50 healthy blue foxes (25 females and 25 males), aged 17 weeks and weighing 6.45 ± 0.36 kg, were selected for this study. No significant differences in body condition were observed among the animals. All foxes were individually housed in outdoor cages (100 cm × 100 cm × 90 cm) at the Jingwen Fur Animal Farm in Jilin City, Jilin Province. The cage dimensions were 100 cm × 100 cm × 90 cm. During the study, all foxes were maintained in a consistent outdoor environment with controlled light, temperature, and humidity conditions. The cages were equipped with accessible water dispensers and feeding troughs to provide ad libitum access to clean water and complete feed. All procedures complied with national regulations and institutional guidelines for animal care and use, ensuring standardized nutritional management throughout the experiment.

### 2.2. Experimental Design and Feeding Scheme

This study employed a completely randomized, single-factor experimental design. A total of 50 healthy blue foxes were randomly assigned to five experimental groups (*n* = 10 per group), with balanced sex distribution (5 males and 5 females per group). Each animal served as an independent experimental unit. The experiment was conducted over a 70-day period. Five isoenergetic diets were formulated as follows: the control group received a basal diet containing 28% CP; the four experimental groups—designated M0 (0% DL-methionine), M1 (0.35%), M2 (0.55%), and M3 (0.75%)—were fed experimental diets containing 22% CP and supplemented with graded levels of DL-methionine, resulting in total methionine concentrations of 0.49%, 0.35%, 0.70%, 0.90%, and 1.10% in the control, M0, M1, M2, and M3, respectively (Table 1). During the first 45 days, animals were fed twice daily at 08:00 and 16:00 (Beijing Time), with ad libitum access to clean drinking water. In the final 25-day phase, feeding frequency was reduced to once daily at 12:00, based on longitudinal behavioral observations indicating decreased spontaneous feeding activity and reduced locomotor activity during snowfall periods. Prior to the formal experiment, all animals underwent a 7-day adaptation period to acclimate to both the experimental diets and housing conditions, thereby improving data stability and experimental reliability.

### 2.3. Body Weight Measurement and Fur Sample Collection

At the beginning of the experiment, the initial body weight (IW) of each blue fox across all experimental groups was recorded. At the end of the trial, final body weight FW was measured to assess growth performance. Pelt length (PL), defined as the distance from the nasal tip to the tail base, was measured using a calibrated ruler. After complete removal of subcutaneous fat from the inner surface of the pelt, pelt weight (PW) was determined using a precision electronic balance to obtain accurate net weight data. Representative dorsal skin samples were aseptically collected from the dorsal region for measurement of guard hair length (GHL) and underfur length (UFL), with care taken to preserve hair shaft integrity and ensure measurement reliability. For both guard hairs and underfur, the length from the skin surface to the hair tip was measured using a calibrated ruler, and the ratio of guard hair length to underfur length (GHL/UFL) was calculated. Skin tissue samples were processed by removing adipose tissue and epidermal debris, fixed in 4% paraformaldehyde, and then subjected to hematoxylin and eosin (H&E) staining and microtome sectioning to enable systematic evaluation of hair follicle development [14].

### 2.4. Collection of Fecal, Urinary, and Feed Samples

Fecal samples were collected daily from blue foxes using the total collection method during the final five days of the experiment [15]. Residual feed was collected from the troughs within 30 min after each feeding. Solid samples, including feces and residual feed, were immediately weighed, oven-dried at 65 °C for 48 h until constant weight was achieved, homogenized using a grinder, and screened through a 2 mm sieve prior to storage. Urine was collected daily via bags positioned beneath the cages; each bag contained 25 mL of 5% sulfuric acid, which was added in advance to minimize ammonia volatilization. For microbial analysis, fresh fecal samples obtained by rectal sampling were flash-frozen in liquid nitrogen, transferred into 5 mL cryovials, and stored at −80 °C until subsequent DNA extraction and 16S rRNA sequencing.

### 2.5. Blood Sample Collection and Analysis

On the final experimental day, following a 12 h fast, blood samples were collected from the hindlimb veins of blue foxes. After collection, the samples were immediately centrifuged at 4000× *g* for 10 min using an Eppendorf 5804 centrifuge (Eppendorf AG, Hamburg, Germany) to obtain serum. Serum concentrations of TP, ALB, HDL-C, LDL-C, glucose (GLU), total cholesterol (TC), triglycerides (TG), alanine aminotransferase (ALT), aspartate aminotransferase (AST), and alkaline phosphatase (ALP) were then measured using a Hitachi 911 clinical chemistry analyzer (Hitachi High-Tech Corporation, Tokyo, Japan) according to the manufacturer’s instructions. The analytical methods were as follows: TP was measured using the biuret assay [16]; ALB, using the bromocresol green (BCG) method [17]; HDL-C and LDL-C, using immunoturbidimetry [18]; GLU, using the glucose oxidase method [19]; TG, using the glycerol-3-phosphate oxidase–peroxidase (GPO-PAP) method [20]; TC, using the enzymatic cholesterol oxidase–peroxidase assay [21]; ALT, AST, and ALP, using malate dehydrogenase-coupled assays [22].

### 2.6. Growth Performance and Nutrient Digestibility Determination

ADG and TWG of blue foxes were calculated from IW and FW. TWG was defined as the difference between body weight measurements taken at the beginning and end of the experiment, and ADG was calculated by dividing TWG by the number of experimental days. DM content was determined according to Method [23]: feed and fecal samples were dried in a forced-air oven at 105 ± 2 °C until constant weight was achieved (weight change ≤ 0.5 mg). N content in feed and fecal samples was analyzed using a LECO FP-528N protein analyzer (LECO Corporation, St. Joseph, MI, USA); CP content was then calculated as CP = N × 6.25 [24]. EE content was determined by Soxhlet extraction [23] using a Büchi B-811 extraction system (Büchi Corporation, Flawil, Switzerland). Briefly, samples underwent 16 extraction cycles with petroleum ether in a 75 °C water bath. OM content was determined by combustion following Method [23]: samples were heated in a muffle furnace from room temperature to 550 °C at 5 °C/min, maintained at 550 °C for 12 h, cooled, and weighed to determine ash content. Calcium (Ca) and phosphorus (P) concentrations in feed were analyzed according to AOAC Method 985.01 [25]: samples were digested in a microwave digestion system with nitric acid–perchloric acid mixture (HNO_3_:HClO_4_, 4:1 *v*/*v*) and quantified by inductively coupled plasma mass spectrometry (ICP-MS) using an Agilent 7900 system (Agilent Technologies, Santa Clara, CA, USA). Amino acid analysis was performed by hydrolyzing feed samples with 6 mol/L hydrochloric acid under nitrogen atmosphere at 110 °C for 24 h, followed by o-phthalaldehyde (OPA) derivatization and separation and quantification using high-performance liquid chromatography (HPLC) [26].

### 2.7. Fecal Microbiota Analysis

Fecal samples (0.2–0.5 g) were retrieved from a −80 °C ultra-low-temperature freezer and homogenized in pre-chilled centrifuge tubes containing lysis buffer. Total genomic DNA was extracted using the Omega Soil DNA Kit (D5635-02, Omega Bio-Tek, Norcross, GA, USA) following the manufacturer’s instructions. DNA fragment integrity was assessed by 0.8% agarose gel electrophoresis (Invitrogen, cat. no. 75510-019), and DNA concentration and purity were quantified using a NanoDrop 2000 spectrophotometer (Thermo Fisher Scientific, Wilmington, DE, USA). PCR amplification targeted the V3–V4 hypervariable region of the bacterial 16S rRNA gene using the following primers: forward primer F (5′-ACTCCTACGGGAGGCAGCA-3′) and reverse primer R (5′-GGACTACHVGGGTWTCTAAT-3′). Amplification was performed on an ABI 2720 Thermal Cycler (Applied Biosystems, Foster City, CA, USA) under the following conditions: initial denaturation at 98 °C for 5 min; 25 cycles of denaturation at 98 °C for 30 s, annealing at 52 °C for 30 s, and extension at 72 °C for 45 s; and a final extension at 72 °C for 5 min. Amplified products were stored at 4 °C pending downstream analysis. The PCR products were separated by 2% agarose gel electrophoresis (Model DYY-6C, Beijing Liuyi Biotechnology Co., Ltd., Beijing, China). Target DNA bands were excised and purified using the Axygen Gel Extraction Kit (Corning Inc., cat. no. AP-GX-50; Bedford, MA, USA). The concentration of double-stranded DNA was then quantified using the Quant-iT PicoGreen dsDNA Assay Kit (Invitrogen, cat. no. P7589; Waltham, MA, USA) on a BioTek FLx800 microplate reader (BioTek Instruments, Inc., Winooski, VT, USA). Libraries were constructed using the Illumina TruSeq Nano DNA LT Library Prep Kit (Illumina, cat. no. 20015965, San Diego, CA, USA). For quality assessment, 1 μL of each library was analyzed on an Agilent 2100 Bioanalyzer (Agilent Technologies, cat. no. G2939BA; Santa Clara, CA, USA) using the Agilent High Sensitivity DNA Kit (Agilent Technologies, cat. no. 5067-4626, Santa Clara, CA, USA). Qualified libraries exhibited a single dominant peak with no detectable adapter dimer peaks.

Paired-end sequencing (2 × 250 bp) was performed on the Illumina NovaSeq 6000 platform using quality-verified libraries. Prior to sequencing, libraries were serially diluted to a final concentration of 2 nM and pooled in equimolar ratios. The pooled library was denatured with 0.1 N NaOH to generate single-stranded DNA templates for cluster amplification and sequencing. Microbiome analysis was conducted using QIIME 2 (version 2022.11), following the standardized 16S rRNA gene amplicon analysis workflow. Raw reads were demultiplexed using the q2-demux plugin; primer sequences were trimmed with Cutadapt (v4.4); and quality filtering, denoising, paired-end read merging, and chimera detection and removal were carried out using the q2-dada2 plugin. Amplicon sequence variants (ASVs) were resolved at 100% sequence identity, and the resulting ASV feature table—including taxonomic assignments and sample-wise abundance counts—was generated for downstream statistical and ecological analyses.

### 2.8. Statistical Analysis

Growth performance, fur quality, macronutrient digestibility, N metabolism, and serum biochemical parameters were analyzed using SPSS Statistics 25.0 software (IBM Corporation, Armonk, NY, USA). Data were subjected to one-way analysis of variance (ANOVA). When significant differences were detected among groups, post hoc comparisons were performed using the least significant difference (LSD) test or Duncan’s multiple range test. Results are expressed as mean ± standard deviation (mean ± SD). Statistical significance was defined as *p* < 0.05.

For microbiome analysis, taxonomic classification and annotation of representative amplicon sequence variants (ASVs) were performed using reference databases including SILVA (version 138.1) [27], Greengenes [28], NCBI [29], and FunGene [30]. Alpha diversity indices—including Chao1, observed ASVs, Shannon, Simpson, Faith’s phylogenetic diversity (PD), Pielou’s evenness, and Good’s coverage—were calculated using QIIME 2 (version 2022.11) and visualized with box plots to display species richness and evenness. Beta diversity was assessed based on weighted and unweighted UniFrac distances computed in QIIME 2 and R, with principal coordinates analysis (PCoA) used to illustrate microbial community dissimilarities. Taxonomic assignment was performed using the classify-sklearn plugin in QIIME 2, followed by refinement through sequence alignment. Relative abundances at the phylum and genus levels were visualized using bar plots in R (version 4.3.1), while compositional profiles across six taxonomic levels were extracted from QIIME 2 and presented as heatmaps.

## 3. Results

### 3.1. Growth Performance

No significant difference in IW was observed among the five experimental groups (*p* > 0.05). Dietary protein restriction significantly reduced FW in the M0 group. With graded methionine supplementation, FW in the M3 group was comparable to that of the control group (*p* > 0.05) but significantly greater than that of the M0 group (*p* < 0.05). Similarly, both TWG and ADG were significantly lower in the M0 group relative to the control group (*p* < 0.05). In contrast, the M3 group exhibited significantly higher TWG and ADG than the M0, M1, and M2 groups (*p* < 0.05) (Table 2).

### 3.2. Fur Quality

The results demonstrated that, following methionine restriction, the M0 group exhibited significantly lower PW, PL, and GHL than the control group (*p* < 0.05). With graded methionine supplementation, PW and GHL in the M1, M2, and M3 groups did not differ significantly from those of the control group (*p* > 0.05), and PL in the M2 and M3 groups was comparable to that of the control group (*p* > 0.05). UFL did not differ significantly among the M2, M3, and control groups (*p* > 0.05), but was significantly greater in both the M2 and M3 groups than in the M0 or M1 groups (*p* < 0.05). The GHL/UFL ratio showed no significant overall difference across groups (*p* > 0.05), yet was significantly higher in the M1 and M2 groups than in the M0 group (*p* < 0.05). Primary hair follicle (PHF) density was not significantly different across all experimental groups (*p* > 0.05). In contrast, secondary hair follicle (SHF) density was significantly lower in the M0 group (*p* < 0.05) and significantly higher in the M2 and M3 groups relative to the M0 group following methionine supplementation (*p* < 0.05). Although no significant difference was observed in the secondary-to-primary hair follicle (S/P) ratio between any experimental group and the control group (*p* > 0.05), the ratio in the M3 group was significantly higher than that in the M0 group (*p* < 0.05) (Table 3).

### 3.3. Nutrient Digestibility

The results showed that methionine supplementation in low-protein diets did not significantly affect DM intake in blue foxes compared with the control group fed a normal-protein diet (*p* > 0.05). DM excretion was significantly higher in the M0 and M1 groups than in the control group (*p* < 0.05), but no significant difference was observed for the M2 and M3 groups compared with the control group (*p* > 0.05). DM digestibility was significantly greater in the M2 and M3 groups than in the control, M0, and M1 groups (*p* < 0.05). OM digestibility was significantly lower in the M0, M1, and M2 groups than in the control group (*p* < 0.05), likely attributable to reduced dietary protein content; however, it was restored to a level comparable to that of the control group in the M3 group (*p* > 0.05). CP digestibility was highest in the M3 group (*p* < 0.05) and significantly greater in the control group than in the M0, M1, and M2 groups (*p* < 0.05). Regarding EE digestibility, the M1, M2, and M3 groups did not differ significantly from the control group, but all were significantly higher than the M0 group (*p* < 0.05) (Table 4).

### 3.4. Nitrogen Metabolism

The results demonstrated that methionine supplementation in low-protein diets had no significant effect on N intake or fecal N excretion in blue foxes compared with the control group fed a normal-protein diet (*p* > 0.05). However, a numerical trend indicated that both parameters decreased as the dietary protein level declined. Urinary N excretion was significantly lower in the M2 and M3 groups than in the control, M0, and M1 groups (*p* < 0.05). The M0 and M1 groups also had lower urinary N than the control group (*p* < 0.05). Conversely, N retention was significantly higher in the M2 and M3 groups than in the other groups (*p* < 0.05) (Table 5).

### 3.5. Serum Biochemical Parameters

The results demonstrated that TP concentrations were significantly lower in the M0 and M1 groups relative to the control group (*p* < 0.05). In contrast, TP concentrations in the M2 and M3 groups exhibited a progressive increase with escalating methionine supplementation, reaching levels statistically indistinguishable from those of the control group at the conclusion of the experiment (*p* > 0.05). ALB concentrations were comparable between the control and M3 groups, both of which were significantly higher than those observed in the M0, M1, and M2 groups (*p* < 0.05). Moreover, ALB concentrations increased in a dose-dependent manner with methionine supplementation, being significantly elevated in the M1 and M2 groups compared with the M0 group (*p* < 0.05). No statistically significant differences were detected in GLU, TC, or TG concentrations across the five experimental groups (*p* > 0.05). HDL concentrations in the M2 and M3 groups did not differ significantly from the control group but were significantly greater than those in the M0 and M1 groups (*p* < 0.05). LDL concentrations were significantly higher in the control group than in all other groups (*p* < 0.05); however, the M2 and M3 groups displayed significantly higher LDL concentrations than the M0 group (*p* < 0.05). ALT concentrations were significantly lower in the M2 and M3 groups than in the control group, yet remained significantly higher than those in the M0 group (*p* < 0.05). AST concentrations in the M2 and M3 groups were not significantly different from the control group but were significantly elevated relative to the M0 group (*p* < 0.05). ALP concentrations in the M3 group were comparable to those in the control group but significantly higher than those in the M0, M1, and M2 groups (*p* < 0.05). Notably, ALP concentrations in the M0, M1, and M2 groups increased incrementally with increasing methionine supplementation (Table 6).

### 3.6. Intestinal Microbiota

**Figure 1:**
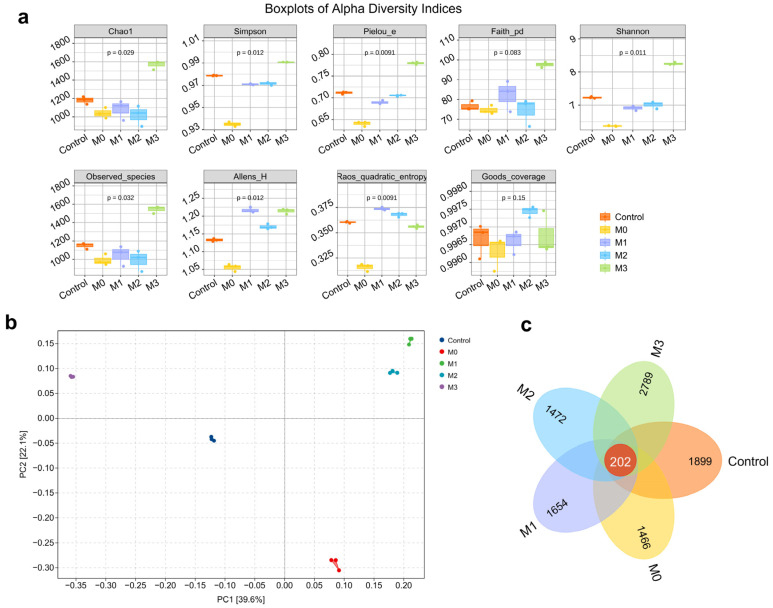
(**a**) Alpha diversity indices of the intestinal microbiota in blue foxes. (**b**) Principal coordinates analysis (PCoA) plot depicting beta diversity among the intestinal microbial communities of blue foxes. (**c**) Venn diagram based on operational taxonomic units (ASVs), illustrating differences in species composition and identifying marker taxa within the intestinal microbiota of blue foxes. The red segment denotes the core microbiota common to all five groups, comprising 202 ASVs (amplicon sequence variants), whereas the remaining numerical values represent the group-specific core microbiota unique to each respective group.

Figure 1a demonstrates that following protein restriction, all alpha diversity indices in the M0 group were significantly lower than those in the control group (*p* < 0.05). Strikingly, the M3 group exhibited significantly higher values than all other groups across seven key indices: Chao1, observed species count, and Faith’s phylogenetic diversity (reflecting richness); Simpson, Pielou’s evenness, Shannon, and Allen’s H indices (reflecting evenness) (*p* < 0.05). Moreover, methionine supplementation produced a dose-dependent increase in both richness-related (Chao1, observed species) and evenness-related (Simpson, Pielou’s evenness, Shannon) indices across the M0–M3 groups. These findings indicate that methionine supplementation enhances both microbial community richness and evenness in a dose-dependent manner.

Figure 1b presents the beta-diversity analysis using principal coordinate analysis (PCoA), demonstrating distinct separation of the M3 group from other experimental groups in the ordination space. The greatest separation was observed between the M3 and M0 groups. In contrast, the M1 and M2 groups formed a more compact cluster, indicating higher similarity in gut microbial community composition between these two groups.

Figure 1c displays a Venn diagram illustrating shared and unique amplicon sequence variants (ASVs) among blue fox groups during the winter fur growth phase. A core microbiome of 202 ASVs was consistently detected across all five groups, reflecting a conserved microbial core. The number of group-specific ASVs was 1899 in the control group, 1466 in the M0 group, 1654 in the M1 group, 1472 in the M2 group, and 2789 in the M3 group, indicating that the M3 group exhibited the highest taxonomic uniqueness.

**Figure 2:**
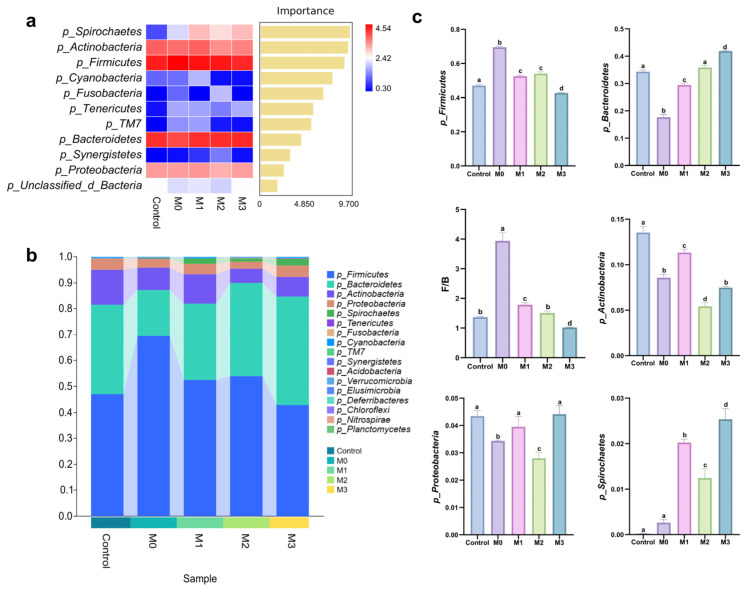
(**a**) Heatmap of the top 20 phylum-level microbial taxa based on average relative abundance in the intestinal microbiota of blue foxes during the fur growth period. (**b**) Bar plot depicting the compositional structure of the phylum-level microbial community. (**c**) Bar plot visualization of phylum-level microbial taxa exhibiting significant inter-group differences and high relative abundance. Values sharing different lowercase letters indicate statistically significant differences (*p* < 0.05).

To further clarify the differences in species composition among samples and demonstrate the trends of abundance distribution in each sample, we constructed a heatmap of species composition using the abundance data of the top 20 phyla ranked by average abundance (Figure 2a). By combining the differentially abundant taxa identified in the heatmap analysis with the feature table after removing single-copy sequences, we achieved a comprehensive visualization of the microbial composition at the phylum level (Figure 2b). Based on the combined results of Figure 2a,b, we conducted a systematic analysis of the six most abundant dominant genera (Figure 2c).

The results revealed that *Firmicutes* and *Bacteroidetes* remained the dominant bacterial phyla across all five experimental groups. The relative abundance of *Firmicutes* was significantly elevated in the M0, M1, and M2 groups compared to the control group (*p* < 0.05), with the greatest increase observed in M0. In contrast, the M3 group—which received the highest methionine supplementation—showed a significant reduction in *Firmicutes* abundance relative to the control (*p* < 0.05). For *Bacteroidetes*, dietary protein restriction led to a significantly lower abundance in the M0 group than in the control (*p* < 0.05), but incremental methionine supplementation resulted in progressive recovery in the M1 and M2 groups. The M2 group did not differ significantly from the control, whereas the M3 group exhibited a higher abundance (*p* < 0.05). Concurrently, the *Firmicutes/Bacteroidetes* (F/B) ratio was markedly elevated in the M0 group compared to all other groups (*p* < 0.05) and decreased dose-dependently with increasing methionine supplementation.

The relative abundance of *Actinobacteria* was significantly reduced in all treatment groups (M0–M3) relative to the control group (*p* < 0.05). Notably, M0 and M3 exhibited comparable abundances—both markedly lower than those in the control and M1 groups (*p* < 0.05) yet significantly higher than that in the M2 group (all *p* < 0.05). For *Proteobacteria*, abundances in the control, M1, and M3 groups were statistically indistinguishable and significantly greater than those in the M0 and M2 groups (*p* < 0.05); moreover, the M2 group displayed significantly lower abundance than the M0 group (*p* < 0.05). Collectively, these results demonstrate non-linear, dose-dependent responses of *Actinobacteria* and *Proteobacteria* to dietary methionine supplementation. Regarding *Spirochaetes,* no significant difference was detected between the control and M0 groups. However, both exhibited significantly lower relative abundance than the M1, M2, and M3 groups (*p* < 0.05), with the M3 group showing the highest level—significantly exceeding those of M1 and M2 (*p* < 0.05).

**Figure 3:**
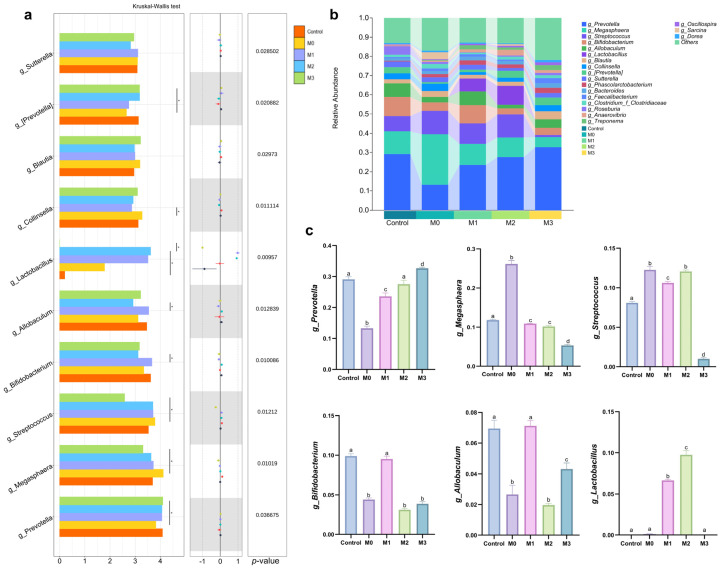
(**a**) Analysis of species differences in the intestinal microbiota at the genus level in blue foxes during the fur growth period. On the left is a bar chart with the horizontal axis representing the corresponding abundance-unit conversion values; in the middle, differently colored dots visualize differences among the corresponding species; on the right are the *p*-values obtained from statistical tests. (**b**) Bar plot depicting the compositional structure of the genera-level microbial community. (**c**) Bar plot visualization of genera-level microbial taxa exhibiting significant inter-group differences and high relative abundance. Values sharing different lowercase letters indicate statistically significant differences (*p* < 0.05).

To further characterize the compositional patterns of high-abundance, genus-level differential taxa, we constructed two visualizations: (1) a genus-level species difference analysis plot illustrating intestinal microbiota variations in blue foxes during the fur growth period (Figure 3a), and (2) a bar chart depicting the composition of the genus-level microbial community structure (Figure 3b). Building on the integrated findings from Figure 3a,b, a systematic analysis was performed on the top six most abundant dominant genera (Figure 3c).

At the genus level, dietary protein restriction significantly reduced the relative abundance of *Prevotella* in the M0 group compared with the control (*p* < 0.05). However, a dose-dependent restoration of *Prevotella* abundance was observed with increasing methionine supplementation: at the M2 level (0.55% methionine), it rebounded to a level comparable to that of the control group; at the highest level (M3, 0.75% methionine), it significantly exceeded that of the control (*p* < 0.05). In contrast, the relative abundance of *Megasphaera* was significantly elevated under protein restriction (M0, *p* < 0.05) but exhibited a monotonic decrease across the methionine supplementation gradient (M1–M3), reaching its lowest level in the M3 group—which was significantly lower than that of the control (*p* < 0.05). Conversely, compared with the control group, the abundance of *Streptococcus* was significantly higher in the M0, M1, and M2 groups (*p* < 0.05), but markedly lower in the M3 group, where it was significantly reduced relative to all other groups (*p* < 0.05). This pattern suggests a biphasic response: initial enhancement under low-protein conditions followed by pronounced suppression at the highest methionine supplementation level. Meanwhile, *Bifidobacterium* abundance exhibited a non-monotonic response: it was significantly reduced under protein restriction (M0, *p* < 0.05), returned to control levels in the M1 group (*p* > 0.05), and decreased again at elevated methionine supplementation levels (M2 and M3, *p* < 0.05).

Compared with the control group, the relative abundance of *Allobaculum* was significantly lower in the M0, M2, and M3 groups (*p* < 0.05). Although the M1 group did not differ significantly from the control group, the overall trend indicated a nonlinear response: the relative abundance of *Allobaculum* initially decreased and then slightly recovered with increasing methionine supplementation levels, suggesting complex regulatory mechanisms under dietary modulation. The relative abundance of *Lactobacillus* was comparable among the control, M0, and M3 groups, with no significant differences. However, it was significantly elevated in the M1 and M2 groups (*p* < 0.05), followed by a significant decrease in the M3 group (*p* < 0.05), resulting in a “rise-then-fall” pattern. This suggests that moderate methionine supplementation enhances *Lactobacillus* proliferation, whereas excessive supplementation may disrupt its ecological niche or metabolic balance.

## 4. Discussion

In this study, blue foxes in the control group were fed a basal diet containing 28% CP—a formulation commonly used in fur-bearing animal production that meets their nutritional requirements during the winter fur growth phase. Due to inconsistencies across previous studies in basal diet composition, feeding phases, experimental duration, and methionine levels—and supported by evidence on N utilization efficiency [13], fur quality [31], and nutrient digestibility [32,33]—we reduced dietary protein to 22% CP and supplemented with four levels of methionine (0%, 0.35%, 0.55%, and 0.75%). Given the lack of systematic data on how reduced protein intake and methionine supplementation jointly affect the intestinal microbiota, this study also examined their impact on the structure and function of the intestinal microbial community in blue foxes.

This study demonstrated that dietary protein reduction in the absence of methionine supplementation significantly impaired growth performance in blue foxes, as evidenced by reductions in FW, TWG, and ADG. Over a 70-day feeding trial, incremental methionine supplementation (0.35%, 0.55%, and 0.75%) led to a dose-dependent improvement in growth parameters. Notably, at the 0.75% supplementation level, FW, TWG, and ADG were restored to values comparable to those of the control group (28% CP), indicating that adequate exogenous methionine can effectively mitigate the adverse effects of reduced dietary protein. These findings are supported by previous experimental evidence: Dahlman T. et al. [34] reported significantly reduced weight gain in blue foxes fed a 15% crude protein diet devoid of supplemental methionine from August to mid-September, compared with animals receiving the P22.5 and P30 diets; Zhang, H. H. et al. [12] further demonstrated that from July to mid-September, the ADG and F/G of minks fed a low-protein diet supplemented with 0.6% methionine were superior to those of the high-protein group. Furthermore, Bunchasak C. et al. [35] attributed this effect to methionine’s role in optimizing dietary amino acid balance and enhancing metabolic efficiency—thereby improving feed conversion efficiency and promoting sustained somatic growth.

Hair is predominantly composed of keratin, the main structural component of the hair matrix [36]. Hair fiber diameter is highly sensitive to dietary methionine levels. Methionine is metabolized to S-adenosylmethionine (SAM), which undergoes hydrolysis to produce homocysteine—a critical intermediate in the biosynthesis of hair proteins [37]. Evidence from previous studies demonstrates that methionine supplementation significantly increases hair fiber diameter and improves underfur quality in Angora rabbits [38]. In minks, adding 0.8% methionine to a low-protein diet markedly enhances fur quality and PW [39]. In this study, increasing methionine supplementation led to dose-dependent improvements in PW and PL, with optimal values achieved at a 0.75% inclusion level. No significant differences in UFL were observed between the control group and groups supplemented with 0.55% or 0.75% methionine, indicating that maximal hair growth was attained within this range. Concurrently, as methionine levels increased from 0.35% to 0.75%, the GHL/UFL ratio progressively decreased, suggesting enhanced fur density and improved pelt compactness. Furthermore, previous studies have demonstrated that methionine supplementation in low-protein diets significantly promotes hair follicle development in Angora rabbits [11]. It is suggested that this effect may be mediated through the regulation of key signaling pathways, including TGFβ-BMP/Shh-Noggin, Wnt10b/β-catenin, EGF, and HGF, thereby promoting hair follicle growth and regeneration under stress conditions [40]. Additionally, transcriptomic analysis of differentially expressed genes in PHF and SHF revealed significant enrichment in methionine and cysteine metabolic pathways [41]. These findings align with the improved hair follicle development observed in the present study, further underscoring the pivotal role of methionine in hair follicle morphogenesis. Collectively, optimizing methionine supply in low-protein diets not only effectively maintains or enhances fur quality but also supports production performance comparable to that of conventional high-protein feeding regimens, highlighting its potential for practical application in animal production.

Apparent nutrient digestibility is a key parameter for assessing nutrient digestion and absorption capacity in blue foxes. Previous studies have demonstrated that supplementation with either DL-methionine or L-methionine in low-protein diets significantly improves the apparent digestibility of EE, whereas supplementation with DL-methionine alone enhances the digestibility of DM and N-free extract (NFE) [32]. Similarly, Blaza et al. [42] reported that increasing dietary methionine supply in growing dogs significantly improved the digestibility of CP and DM. In the present study, the 0.75% methionine group exhibited the highest CP digestibility among all treatment groups. Furthermore, EE digestibility increased progressively with increasing methionine supplementation levels, and the highest digestibility values for DM and OM were observed in the groups receiving the highest methionine doses. These findings are not only consistent with previous studies but also strengthen the evidence for the beneficial effect of methionine in improving nutrient digestion efficiency. Overall, the results demonstrate that supplementing low-protein diets with methionine effectively enhances the digestibility of key nutrients in blue foxes, underscoring its potential for application in dietary management strategies.

The inclusion of methionine in low-protein diets significantly influences N metabolism. Although no significant differences were observed in N intake or fecal N content among treatment groups following amino acid supplementation, urinary N excretion was markedly reduced in the 0.55% and 0.75% methionine-supplemented groups. In contrast, N retention rates were significantly higher in the 0.55% and 0.75% methionine groups. These results suggest that elevated dietary methionine levels enhance the metabolic efficiency of protein utilization. Urea represents the primary form of N in urine, and its rapid hydrolysis into ammonia—followed by volatilization into the atmosphere—substantially contributes to environmental pollution [43]. According to Allison [44], methionine supplementation reduces urinary urea N excretion and increases the ammonia N-to-urea N ratio in adult dogs, demonstrating a nitrogen-sparing effect. Moreover, the strategy of reducing dietary CP content to mitigate N emissions has been consistently supported by multiple studies [45,46]. Collectively, these findings indicate that supplementing low-protein diets with methionine not only decreases N excretion but also improves the efficiency of dietary protein utilization.

Serum biochemical parameters serve as valuable indicators for assessing the health status of blue foxes. TP and ALB play essential roles in hormone regulation, fatty acid transport, maintenance of vascular permeability, and colloid osmotic pressure in the bloodstream [47,48]. Levels of HDL and LDL are not only associated with a reduced risk of atherosclerosis-related mortality but also confer protective effects, including anti-apoptotic, anti-thrombotic, and anti-infective properties [49,50,51]. ALT and AST are well-established markers of hepatic function [52]. The liver performs central physiological roles in protein metabolism, and its functional integrity is highly dependent on nutritional status, particularly dietary protein and amino acid availability [53]. ALP is primarily linked to biliary system function [52]. In this study, TP and ALB levels progressively increased with higher methionine supplementation in low-protein diets, indicating that methionine enhances protein utilization efficiency and overall nutritional status, thereby contributing to improved health outcomes in blue foxes. Concurrently, the observed trends in HDL and LDL levels further support this interpretation. Notably, ALT and AST levels in the 0.55% and 0.75% methionine groups did not differ significantly from those in the control group, suggesting that at these supplementation levels, the liver maintains normal protein metabolic activity without evidence of hepatocellular injury or dysfunction. Furthermore, ALP levels in the high-methionine groups were comparable to those in the control group, whereas a significant reduction was observed in the low-methionine group, implying that prolonged feeding of low-protein diets with inadequate methionine supply may impair normal biliary function [52].

The intestinal microbiota of animals undergoes dynamic alterations in response to age and dietary variation [54]. Specifically, α-diversity reflects species richness, evenness, and diversity within a single habitat [55], whereas β-diversity is used to evaluate differences in species composition among microbial communities or along environmental gradients [56]. The findings of this study demonstrate that reducing dietary protein levels results in a decrease in microbial α-diversity, whereas a gradual increase in methionine intake correlates with a progressive enhancement of α-diversity. Notably, under conditions of reduced dietary protein, the intestinal microbial communities of the 0.35% and 0.55% methionine supplementation groups exhibit tighter clustering along the principal coordinate axes, indicating a higher degree of structural similarity. To further characterize the dynamic shifts in microbial community structure, Venn diagram analysis based on shared and unique operational taxonomic units (ASVs) revealed that, under low-protein feeding conditions, the 0% methionine group harbored the lowest number of unique bacterial taxa, whereas the 0.75% methionine group exhibited the highest number of unique taxa. This observation suggests that elevated methionine levels may facilitate the selective enrichment or niche expansion of specific bacterial clades. Collectively, these findings indicate that increasing methionine supplementation under protein-restricted dietary regimens drives substantial restructuring of the blue fox intestinal microbiota. Although the 0.75% supplementation level induced the most pronounced deviation from the baseline microbial state, it concurrently yielded the maximal enhancement of microbial richness and diversity.

This study employed species composition heatmaps and taxonomic annotation to characterize microbial abundance and community composition at the phylum level across the five experimental groups. Our findings revealed that the intestinal microbiota of blue foxes was predominantly composed of *Firmicutes*, *Bacteroidetes*, *Actinobacteriota*, *Proteobacteria*, and *Spirochaetes*, collectively accounting for 99.30% of the total microbial community. Previous research has established a close association between the F/B ratio and intestinal homeostasis [57]. Notably, *Firmicutes* are primarily Gram-positive bacteria [58], whereas *Bacteroidetes* are predominantly Gram-negative and harbor an extensive repertoire of carbohydrate-degrading enzyme-encoding genes, enabling the breakdown of dietary polysaccharides [59,60]. The control group had an F/B ratio of 1.37, compared to 3.93, 1.78, 1.51, and 1.02 in the 0%, 0.35%, 0.55%, and 0.75% methionine groups, respectively. Although a lower F/B ratio is associated with higher obesity risk [61,62], blue foxes require controlled fat accumulation during winter fur growth to meet energy and developmental needs. As methionine supplementation increased, *Bacteroidetes* abundance rose progressively. Given their genetic capacity for carbohydrate metabolism [60], this suggests that methionine enhances dietary carbohydrate degradation and utilization through selective enrichment of *Bacteroidetes*, thereby improving nutrient absorption and supporting optimal growth. *Actinobacteriota* abundance decreased under protein restriction. This decline—especially in *Bifidobacterium*—is often linked to high-fat, low-fiber diets [63] and impaired gut barrier function [64]. In contrast, *Proteobacteria* are a known marker of dysbiosis [65], typically increasing when *Firmicutes* abundance and overall microbial diversity decline, as seen in inflammatory bowel disease (IBD) [66]. Notably, in the 0.75% methionine group, *Proteobacteria* abundance did not differ significantly from that of the controls, indicating no major microbial imbalance. *Spirochaetota* abundance was similar in the control and 0% groups but increased significantly in the 0.35%, 0.55%, and 0.75% groups, rising in a dose-dependent manner and peaking at 0.75%. This suggests that methionine specifically promotes *Spirochaetota* enrichment, although its physiological impact requires further study.

At the genus level, the intestinal microbiota of blue foxes is dominated by *Prevotella*, *Megasphaera*, *Streptococcus*, *Bifidobacterium*, *Allobaculum*, and *Lactobacillus*, collectively accounting for 60.99% of the total intestinal microbial community. *Prevotella* plays a critical role in enhancing carbohydrate, lipid, and amino acid metabolism in animals [67,68,69]. A reduction in dietary protein content significantly decreased *Prevotella* abundance; however, abundance gradually recovered with increasing methionine supplementation: at 0.55% methionine, *Prevotella* levels were comparable to those of the control group, and at 0.75%, they were significantly higher. Given that *Prevotella* abundance is associated with host fat deposition [70] and certain *Prevotella* species in the gastrointestinal tract efficiently degrade complex carbohydrates [71,72], we hypothesize that increased methionine intake enhances carbohydrate utilization and fat absorption by promoting *Prevotella* abundance. Under low-protein conditions, the abundance of the genus *Megasphaera* increased significantly, but it gradually declined with increasing methionine supplementation. Studies have shown that *Megasphaera* elsdenii, a representative species of this genus, utilizes lactic acid and produces butyrate [73]. Butyrate promotes intestinal mucosal repair and development by stimulating epithelial cell differentiation and proliferation, thereby accelerating recovery after injury [74]. Thus, reduced protein intake may induce mild intestinal dysfunction, triggering compensatory enrichment of *Megasphaera* as part of an adaptive response. With increased methionine supplementation, intestinal homeostasis and tissue integrity are restored, reducing the necessity for such microbial shifts and resulting in decreased abundance of *Megasphaera*. Notably, during protein restriction with methionine supplementation, the abundance of *Streptococcus* exhibited a biphasic pattern—initially increasing, followed by a sharp decline, particularly at the 0.75% methionine supplementation level. Previous studies have demonstrated that certain *Streptococcus* species, such as *Streptococcus* agalactiae in fish, exhibit pathogenic potential and are associated with meningitis, sepsis, ascites, and anorexia [75]. Therefore, the initial increase in *Streptococcus* abundance may indicate microbial dysbiosis or elevated infection risk, whereas high-dose methionine appears to modulate the gut environment, suppress pathogen overgrowth, and regulate microbial composition. Under low-protein conditions, however, *Bifidobacterium*, *Allobaculum*, and *Lactobacillus* displayed distinct response patterns: their abundances fluctuated irregularly rather than following a consistent trend with increasing methionine supplementation. This suggests that the regulation of these genera involves complex, multifactorial mechanisms—potentially influenced by host metabolic status, microbial interactions, and substrate availability. The underlying regulatory mechanisms remain unclear and warrant further investigation. *Lactobacillus* is widely recognized as a beneficial gut commensal that contributes to the maintenance of intestinal homeostasis by reinforcing the epithelial barrier, modulating immune responses, and inhibiting pathogen colonization [76]. Notably, in the present study, *Lactobacillus* was exclusively detected in the groups supplemented with 3.5% and 5.5% methionine, with no enrichment observed in the other groups. This selective occurrence suggests that its growth and colonization are contingent upon specific nutritional or metabolic conditions, highly sensitive to methionine levels, and may reflect a threshold effect in ecological adaptability. In conclusion, the present study demonstrates that reducing dietary CP levels while supplementing with methionine not only significantly alters the diversity and richness of the intestinal microbiota in blue foxes but also exerts a pronounced regulatory effect on dominant bacterial taxa at both the phylum and genus levels. Nevertheless, the underlying mechanisms driving shifts in certain microbial populations warrant further investigation.

## 5. Conclusions

This study demonstrates that dietary methionine supplementation in low-protein diets significantly reduces nitrogen emissions while maintaining growth performance and fur quality comparable to high-protein diets, without compromising animal health. Notably, although the mechanisms underlying certain intestinal microbial changes require further investigation, both gut microbiota diversity and community structure were significantly improved. Furthermore, during the winter fur-growth phase, the optimal methionine supplementation level was determined to be 0.75% DM, corresponding to a dietary methionine requirement of 1.1%. Collectively, these findings highlight the crucial role of methionine in enhancing nutrient utilization and promoting hair follicle development, thereby providing a cost-effective and eco-friendly alternative to high-protein diets.

## Figures and Tables

**Table 1:** Ingredients and chemical composition of diets (dry matter basis).

Items	Diets				
	Control	M0	M1	M2	M3
Ingredients					
Extruded corn	25.05	43.89	43.54	43.34	43.08
Soybean meal	25.30	12.50	12.50	12.50	12.56
Bone and meat meal	8.25	8.9	8.9	8.9	8.9
Corn germ meal	20.75	13.89	13.89	13.89	13.89
Fish meal	9.20	7.9	7.9	7.9	7.9
Soybean oil	10	10	10	10	10
Salt	0.3	0.3	0.3	0.3	0.3
Premix ^(1)^	1	1	1	1	1
Lys	0.10	0.51	0.51	0.51	0.51
Met	0.05	0	0.35	0.55	0.75
Thr	0	0.24	0.24	0.24	0.24
Arg	0	0.59	0.59	0.59	0.59
Ile	0	0.28	0.28	0.28	0.28
Total	100	100	100	100	100
Nutrient levels ^(2)^					
CP (%)	28.02	22.06	22.03	22.01	22.02
EE (%)	12.54	12.39	12.38	12.38	12.38
Ash	7.29	6.36	6.36	6.36	6.36
Ca	1.28	1.23	1.23	1.23	1.23
P	1.02	0.98	0.98	0.98	0.98
Lys (%)	1.66	1.66	1.66	1.66	1.66
Met (%)	0.49	0.35	0.70	0.90	1.10
Thr (%)	1.04	1.04	1.04	1.04	1.04
Arg (%)	1.98	1.98	1.98	1.98	1.98
Ile (%)	1.12	1.12	1.12	1.12	1.12
ME (MJ/kg)	14.49	14.48	14.43	14.41	14.37

Note: ^(1)^ The premix contained per kg: Mixed vitamins 16 g; nicotinic acid 4000 mg; pantothenic acid 1200 mg; alkaloid 20 mg; folic acid 80 mg; choline 30,000 mg; Fe 8200 mg; Cu 800 mg; Mn 1200 mg; Zn 5200 mg; I 50 mg; Se 20 mg; Co 50 mg. ^(2)^ Nutritional levels presented herein are measured values.

**Table 2:** Effects of methionine supplementation in low-protein diets on growth performance of blue foxes.

Items	Groups				
	Control	M0	M1	M2	M3
IW (kg)	6.17 ± 0.06	6.58 ± 0.32	6.55 ± 0.31	6.62 ± 0.28	6.35 ± 0.64
FW (kg)	8.46 ± 0.47 ^ab^	7.86 ± 0.14 ^a^	8.38 ± 0.32 ^ab^	8.65 ± 0.36 ^ab^	9.00 ± 0.90 ^b^
TWG (kg)	2.29 ± 0.45 ^bc^	1.28 ± 0.20 ^a^	1.83 ± 0.20 ^ab^	2.03 ± 0.58 ^ab^	2.87 ± 0.58 ^c^
ADG (g)	33.73 ± 6.59 ^bc^	18.78 ± 2.90 ^a^	26.94 ± 2.65 ^ab^	29.90 ± 8.52 ^ab^	42.20 ± 8.53 ^c^

Note: IW: Initial Weight; FW: Final Weight; TWG: Total Weight Gain; ADG: Average Daily Gain. In the same row, values with no superscript letters or the same lowercase superscript letters indicate no significant difference (*p* > 0.05), whereas different lowercase superscript letters indicate a significant difference (*p* < 0.05).

**Table 3:** Effects of methionine supplementation in low-protein diets on fur quality of blue foxes.

Items	Groups				
	Control	M0	M1	M2	M3
PW (g)	1.87 ± 0.14 ^b^	1.54 ± 0.87 ^a^	1.73 ± 0.06 ^b^	1.79 ± 0.93 ^b^	1.94 ± 0.17 ^b^
PL (cm)	107.75 ± 0.96 ^b^	102.00 ± 2.00 ^a^	102.25 ± 2.06 ^a^	107.00 ± 0.82 ^b^	109.00 ± 0.82 ^b^
GHL (mm)	6.83 ± 0.43 ^b^	5.05 ± 0.33 ^a^	5.78 ± 0.81 ^ab^	6.93 ± 1.64 ^b^	6.13 ± 0.48 ^ab^
UFL (mm)	5.04 ± 0.21 ^ab^	4.40 ± 0.54 ^a^	4.58 ± 0.75 ^a^	5.63 ± 1.24 ^b^	5.44 ± 0.39 ^b^
GHL/UFL	1.23 ± 0.03 ^ab^	1.16 ± 0.07 ^b^	1.26 ± 0.03 ^a^	1.27 ± 0.04 ^a^	1.21 ± 0.07 ^ab^
PHF (mm^−2^)	8.00 ± 0.82	7.75 ± 0.50	8.00 ± 0.10	8.00 ± 0.82	7.75 ± 0.50
SHF (mm^−2^)	863.25 ± 32.16 ^a^	761.00 ± 27.60 ^b^	805.50 ± 60.30 ^ab^	860.75 ± 29.48 ^a^	866.25 ± 28.28 ^a^
S/P	108.40 ± 7.32 ^ab^	98.54 ± 7.86 ^b^	100.69 ± 7.54 ^ab^	108.25 ± 8.86 ^ab^	112.00 ± 5.16 ^a^

Note: PW: Pelt weight; PL: Pelt length; GHL: Guard hair length; UFL: Underfur length; GHL/UFL: Guard hair/underfur; PHF: Primary hair follicle; SHF: Secondary hair follicle; S/P: Secondary/Primary. In the same row, values with no superscript letters or the same lowercase superscript letters indicate no significant difference (*p* > 0.05), whereas different lowercase superscript letters indicate a significant difference (*p* < 0.05).

**Table 4:** Effects of methionine supplementation in low-protein diets on nutrient digestibility in blue foxes.

Items	Groups				
	Control	M0	M1	M2	M3
DM intake (g/d)	412.93 ± 10.90	411.21 ± 4.28	414.83 ± 12.70	406.63 ± 5.40	408.70 ± 6.00
DM output (g/d)	161.09 ± 4.23 ^ab^	168.07 ± 2.22 ^a^	162.50 ± 5.98 ^a^	154.24 ± 0.89 ^b^	154.39 ± 3.37 ^b^
DM (%)	60.99 ± 0.01 ^b^	59.13 ± 0.28 ^a^	60.75 ± 0.25 ^b^	62.07 ± 0.28 ^c^	62.22 ± 0.28 ^c^
OM (%)	65.87 ± 0.34 ^c^	62.07 ± 0.21 ^a^	62.16 ± 0.25 ^a^	64.74 ± 0.61 ^b^	65.72 ± 0.56 ^c^
CP (%)	62.87 ± 2.08 ^b^	61.09 ± 2.70 ^a^	60.66 ± 1.50 ^a^	60.57 ± 2.19 ^a^	64.04 ± 0.44 ^c^
EE (%)	85.79 ± 2.02 ^b^	81.94 ± 0.14 ^a^	84.45 ± 2.92 ^b^	85.06 ± 2.62 ^b^	84.73 ± 1.29 ^b^

Note: DM: dry matter; CP: crude protein; EE: ether extract; OM: organic matter. In the same row, values with no superscript letters or the same lowercase superscript letters indicate no significant difference (*p* > 0.05), whereas different lowercase superscript letters indicate a significant difference (*p* < 0.05).

**Table 5:** Effects of methionine supplementation in low-protein diets on nitrogen metabolism in blue foxes.

Items	Groups				
	Control	M0	M1	M2	M3
N intake (g/d)	12.93 ± 0.90	12.79 ± 0.46	12.84 ± 0.65	12.64 ± 0.49	12.71 ± 0.67
Fecal N (g/d)	5.65 ± 0.39	4.98 ± 0.5	5.50 ± 0.35	4.89 ± 0.70	4.57 ± 0.86
Urine N (g/d)	4.51 ± 0.42 ^c^	3.01 ± 0.20 ^a^	2.9 ± 0.18 ^a^	2.43 ± 0.76 ^b^	2.73 ± 0.05 ^b^
N retention (g/d)	5.07 ± 0.56 ^b^	4.81 ± 0.80 ^a^	4.89 ± 0.33 ^a^	5.23 ± 0.58 ^c^	5.41 ± 0.96 ^c^

Note: In the same row, values with no superscript letters or the same lowercase superscript letters indicate no significant difference (*p* > 0.05), whereas different lowercase superscript letters indicate a significant difference (*p* < 0.05).

**Table 6:** Effects of methionine supplementation in low-protein diets on serum biochemical parameters in blue foxes.

Items	Groups				
	Control	M0	M1	M2	M3
TP (g/L)	62.47 ± 1.80 ^a^	57.30 ± 2.18 ^b^	58.27 ± 0.15 ^bc^	59.97 ± 1.17 ^abc^	60.17 ± 0.98 ^ab^
ALB (g/L)	34.33 ± 0.23 ^a^	31.47 ± 0.23 ^b^	32.60 ± 0.52 ^c^	33.33 ± 0.15 ^c^	35.00 ± 0.78 ^a^
GLU (mmol/L)	5.63 ± 0.33	5.02 ± 0.11	5.22 ± 0.46	5.38 ± 0.95	5.49 ± 0.87
TC (mmol/L)	4.03 ± 0.13	3.96 ± 0.18	3.91 ± 0.67	3.82 ± 0.35	3.91 ± 0.44
TG (mmol/L)	0.46 ± 0.12	0.49 ± 0.03	0.40 ± 0.107	0.49 ± 0.02	0.55 ± 0.06
HDL (mmol/L)	3.54 ± 0.04 ^a^	2.65 ± 0.17 ^b^	2.89 ± 0.15 ^b^	3.30 ± 0.17 ^a^	3.44 ± 0.04 ^a^
LDL (mmol/L)	0.22 ± 0.03 ^a^	0.12 ± 0.01 ^b^	0.14 ± 0.01 ^bc^	0.17 ± 0.02 ^c^	0.16 ± 0.02 ^c^
ALT (U/L)	177.13 ± 7.32 ^a^	142.43 ± 2.92 ^b^	149.60 ± 5.16 ^bc^	157.50 ± 4.43 ^c^	152.3 ± 3.12 ^c^
AST (U/L)	56.30 ± 4.20 ^a^	43.87 ± 3.1 ^b^	50.80 ± 4.11 ^ab^	51.77 ± 4.24 ^a^	57.63 ± 3.79 ^a^
ALP (U/L)	187.93 ± 0.51 ^a^	141.13 ± 10.69 ^c^	151.53 ± 4.86 ^c^	164.20 ± 2.14 ^b^	177.56 ± 8.80 ^a^

Note: TP: total protein; ALB: albumin; HDL: high-density lipoprotein; LDL: low-density lipoprotein; GLU: glucose; TC: total cholesterol; TG: triglyceride; ALT: Alanine aminotransferase AST: Aspartate aminotransferase; ALP: Alkaline phosphatase. In the same row, values with no superscript letters or the same lowercase superscript letters indicate no significant difference (*p* > 0.05), whereas different lowercase superscript letters indicate a significant difference (*p* < 0.05).

## Data Availability

Data may be provided upon request to the corresponding author (zhangaiwu@jlau.edu.cn).

## Abbreviations

IW, Initial Weight; FW, Final Weight; TWG, Total Weight Gain; ADG, Average Daily Gain; PW, Pelt weight; PL, Pelt length; GHL, Guard hair length; UFL, Underfur length; GHL/UFL, Guard hair/underfur; PHF, Primary hair follicle; SHF, Secondary hair follicle; S/P, Secondary/Primary; N, nitrogen; F/B, Firmicutes/Bacteroidetes; DM, dry matter; CP, crude protein; EE, ether extract; OM, organic matter; TP, total protein; ALB, albumin; HDL, high-density lipoprotein; LDL, low-density lipoprotein; GLU, glucose; TC, total cholesterol; TG, triglyceride; ALT, Alanine aminotransferase AST, Aspartate aminotransferase; ALP, Alkaline phosphatase; M0, with 0% methionine; M1, with 0.35% methionine; M2, with 0.55% methionine; M0, with 0.75% methionine; F/G, feed-to-gain ratio.
